# Improved Detection of Acute Coronary Occlusion Myocardial Infarction by an Artificial Intelligence Electrocardiogram Model in Swedish Emergency Departments

**DOI:** 10.1016/j.acepjo.2026.100473

**Published:** 2026-08-14

**Authors:** Thomas Lindow, Axel Nyström, Jakob Lundager Forberg, Arash Mokhtari, Anders Björkelund, Robert Herman, H. Pendell Meyers, Stephen W. Smith, Ulf Ekelund

**Affiliations:** 1 Respiratory Medicine, Allergology, and Palliative Medicine, Department of Clinical Sciences Lund, Lund University, Lund, Sweden; 2 Department of Medicine, Region Kronoberg, Växjö Central Hospital, Växjö, Sweden; 3 Department of Laboratory Medicine, Lund University, Lund, Sweden; Computational Science for Health and Environment (COSHE), Centre for Environmental and Climate Science, Lund University, Lund, Sweden; 4 Department of Emergency Medicine, Helsingborg Hospital, Helsingborg, Sweden; 5 Department of Clinical Sciences Lund, Lund University, Lund, Sweden; 6 Cardiology, Department of Clinical Sciences Lund, Lund University, Lund, Sweden; 7 Department of Cardiology, Skåne University Hospital, Lund, Sweden; 8 Computational Science for Health and Environment (COSHE), Centre for Environmental and Climate Science, Lund University, Lund, Sweden; 9 Cardiovascular Centre Aalst, AZORG Hospital, Aalst, Belgium; 10 Powerful Medical, Bratislava, Slovakia; 11 Department of Emergency Medicine, Carolinas Medical Center, Charlotte, North Carolina, USA; 12 Faculty Emergency Physician, Hennepin County Medical Center; Professor of Emergency Medicine, University of Minnesota, Minneapolis, Minnesota, USA; 13 Emergency Medicine, Department of Clinical Sciences Lund, Lund University, Lund, Sweden; 14 Department of Emergency Medicine, Skåne University Hospital, Lund, Sweden

**Keywords:** acute coronary occlusion, diagnostic accuracy, acute myocardial infarction, ST elevation, ST depression, artificial intelligence

## Abstract

**Objectives:**

The Queen of Hearts (QoH) ECG artificial intelligence model has demonstrated improved sensitivity for detecting occlusion myocardial infarction (OMI) compared with STEMI criteria, but further validation is needed. We aimed to evaluate QoH's diagnostic performance in patients with chest pain at Swedish emergency departments (EDs).

**Methods:**

This retrospective analysis included consecutive patients with chest pain at the ED from the ESC-TROP study (2017-2018). Patients transferred directly from the prehospital setting to the coronary care unit were not included. OMI classification was based on angiographic data and expert adjudication. QoH, conventional STEMI criteria, and the Glasgow ECG Analysis Algorithm were applied to all cases. In addition, extended STEMI criteria incorporating additional ECG leads (-V1, -V2, -V3, -aVL, -aVR, and -III) and OMI criteria for left bundle branch block (modified Sgarbossa criteria) and left ventricular hypertrophy (ST elevation V1-V3 ≥0.25 of R and S) were applied.

**Results:**

Among 24,511 patients (mean age 59 ± 19 years, 52% male), 467 (1.9%) had OMI. QoH achieved higher sensitivity than STEMI criteria (52% [47 to 57] vs 23% [19 to 27]), similar specificity (99% [99 to 99] vs 98% [98 to 98]), higher positive predictive value (51% [47 to 54] vs 17% [15 to 20]), and similar negative predictive value (99% [99 to 99] vs 98 [98 to 99]). The Glasgow algorithm obtained 32% (28 to 37) sensitivity, 98% (98 to 98) specificity, 26% (23 to 30) PPV, and 99% (99 to 99) NPV, and corresponding number for the extended criteria were 41% (36 to 45), 95% (95 to 96), 14% (12 to 15), and 99% (99 to 99).

**Conclusion:**

In ED patients with chest pain, QoH improved sensitivity in OMI detection compared with currently available ECG criteria, with similar specificity.

**The Bottom Line**

Conventional electrocardiographic criteria are significantly limited in detecting acute coronary occlusions leading to myocardial infarction, potentially delaying life-saving treatment. We applied an artificial intelligence electrocardiogram model—Queen of Hearts—to approximately 25,000 patients with chest pain presenting to 5 Swedish emergency departments. Among 467 patients with an occlusive myocardial infarction, Queen of Hearts detected 52% compared with 23% with standard ST-elevation myocardial infarction criteria, while maintaining high specificity. This study, one of the largest real-world validations of this technology, suggests that artificial intelligence-powered electrocardiogram analysis has the potential to reduce the number of missed acute coronary occlusions in emergency care.

## 1 Introduction

### 1.1 Background

Current ECG criteria that indicate the need for emergent reperfusion therapy—ie, ST-elevation myocardial infarction (STEMI) criteria—are confined to the presence of ST-segment elevation.^1^^,^^2^ Although these criteria provide clear guidance for clinicians in the identification of patients with occlusion myocardial infarction (OMI), their sensitivity is suboptimal.^3^ For example, in patients with non-ST elevation myocardial infarction (NSTEMI) 25% have been reported to have an acute coronary occlusion as defined by thrombolysis in myocardial infarction (TIMI)-0 flow, and 34% as defined by TIMI 0/1 flow.^4^, ^5^, ^6^ Moreover, a meta-analysis of the only 3 studies to ever evaluate the sensitivity of the STEMI criteria for OMI showed 43% sensitivity.^3^ The failure to meet STEMI criteria in such cases often leads to delays in reperfusion therapy, which is associated with a significant increase in both short-term and long-term mortality.^4^ Also, even in STEMI cases, ST elevation may be absent in the early stages of acute coronary occlusion, and hyperacute T waves may be the only sign of occlusion.^7^, ^8^, ^9^, ^10^ On the other hand, a significant proportion of acute catheterization laboratory activations for suspected STEMI reveal no culprit lesion or a nonischemic cause for the ST elevation.^11^^,^^12^ Thus, current STEMI criteria are limited both regarding sensitivity and specificity for OMI.^13^^,^^14^

### 1.2 Importance

In recent years, artificial intelligence (AI) using deep convolutional neural networks has shown substantial capability in detecting valuable electrocardiographic information,^15^^,^^16^ not necessarily detectable by the human eye. A novel ECG AI model, named Queen of Hearts (QoH; Powerful Medical, Bratislava) has been shown to improve sensitivity compared with STEMI criteria^16^ and to decrease false positive activations.^17^ QoH was developed from over 18,000 ECGs to detect OMI using angiographic outcomes as reference. In a case-control external validation, it achieved a two-fold higher sensitivity at equal specificity to traditional STEMI criteria in detecting angiographic evidence of OMI.^16^ However, external validation in large datasets with consecutive patients with chest pain is needed. In addition, a comparison with ECG criteria encompassing STEMI equivalent ECG patterns has not been performed. Also, even though patients with STEMI ECG changes often are transferred directly to catheterization laboratories, therefore bypassing the ED, the vast majority of chest pain patients still present through the ED.^14^^,^^18^ While patients with ECG changes strongly indicative of OMI may go directly to the cath lab, the remaining OMI cases presenting to the ED may pose a significant challenge to both human and computer-based ECG interpretations.

### 1.3 Goals of This Investigation

Therefore, we aimed to evaluate the diagnostic performance of QoH in detecting OMI among this low risk unselected population with chest pain at multiple Swedish emergency departments (ED), and compare its performance to conventional STEMI criteria, extended STEMI criteria including STEMI equivalents, and to algorithmic computer-based interpretations.

## 2 Methods

### 2.1 Study Population

This was a retrospective diagnostic accuracy study nested within the prospective cohort of the ESC-TROP study (Effectiveness and Safety of a Clinical Assessment and 0h/1h Troponin Rule-Out Protocol) which included all patients presenting to 5 EDs in southern Sweden with a primary complaint of nontraumatic chest pain between February 01 and November 30 in both 2017 and 2018.^19^ We used the STARD reporting guideline^20^ to draft the manuscript, and the STARD reporting checklist when editing, included in supplements – when editing (Supplements). This study was approved by the Regional Ethics Review Board without the need for active patient consent.

### 2.2 Selection of Participants

For patients who had multiple visits due to chest pain during the study period, only the first visit was included for analysis. According to clinical routine, patients with STEMI that are identified prehospitally are transferred directly to the catheterization laboratory or the coronary care unit, ie, bypassing the ED. Thus, these patients were not included in this study. Patients with STEMI who did present at the ED were included in this study. Patients without a recorded troponin value, no Swedish Social Security number, who left the ED against recommendations, or had an ECG of unacceptable quality at the ED visit were excluded. In case of several ECGs being recorded for the same patient, only the first was included. Troponin values were recorded according to clinical routine, and were not necessarily followed to peak.

Data on diagnoses of acute myocardial infarction (AMI) or revascularization within 30 days from ED arrival were retrieved from regional electronic health records, the SWEDEHEART register^21^ and the Swedish national patient register.^22^ Angiography results were obtained from the Swedish Coronary Angiography and Angioplasty Register (SCAAR).^21^ In the SCAAR register, patients may be registered as having an acute coronary occlusion, but there is no clear definition and no information on TIMI flow, and complementary information/images from the local patient angiography records were evaluated if needed.

### 2.3 OMI Definition and Adjudication

**Figure 1:**
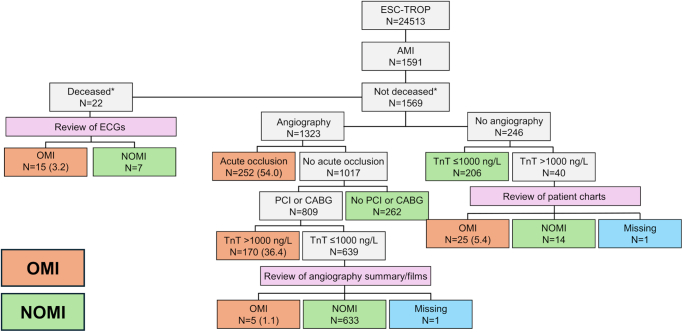
Flow chart of the annotation process. Red boxes denote occlusion myocardial infarction (OMI) cases, green boxes denote nonocclusion myocardial infarction (NOMI) cases, and blue boxes—cases with missing information (n = 2). The percentage of OMI identified in each step is noted within parentheses for OMI cases (red). ∗Among the 1591 patients with acute myocardial infarction (AMI), 22 patients died within 3 days from admission.

The OMI adjudication aimed to classify all cases as either OMI, nonocclusion myocardial infarction (NOMI), or no AMI using a structured, multistep annotation process, described in detail in Fig 1. The annotation process was performed prior to applying any of the ECG methods evaluated in this study.

#### 2.3.1 Patients with AMI Who Underwent Coronary Angiography

Among patients who underwent coronary angiography and were diagnosed with AMI, classification was based on either (1) presence of an angiographically documented acute complete coronary occlusion in the SCAAR registry (N = 252), (2) presence of a significant stenosis resulting in revascularization in combination with a large hs-TnT increase (≥1000 ng/L^23^) (N = 170), or (3) a detailed reevaluation of angiographic reports or films among patients who either were not revascularized or had hs-TnT <1000 ng/L. An emergency physician (JLF) evaluated these angiography reports and identified OMI cases based on the presence of an acute culprit lesion and a TIMI flow of 0 or 1. If a report indicated reduced flow without a definitive TIMI classification, a cardiologist (AM) and an emergency physician (JLF) jointly assessed whether TIMI flow 0 or 1 was present, and this included reevaluation of the angiography films if necessary (OMI cases N = 170). Both physicians were blinded to the ECG results during the annotation process.

#### 2.3.2 Patients with AMI who did not undergo coronary angiography

For AMI patients who did not undergo angiography, the classification followed the criteria established by Meyers et al, which have been used in previous validation studies on ECG criteria for detection of OMI.^24^, ^25^, ^26^ According to these criteria, TnT values ≥1000 ng/L in a patient with highly suspected ACS is strongly indicative of an occluded coronary artery at the time of ECG recording.^23^^,^^27^, ^28^, ^29^ Using these criteria, 25 additional OMI cases were identified.

OMI classification was performed blinded to ECG information, except for patients with AMI who died within 3 days of the index ECG and did not undergo coronary angiography (N = 22). In these cases, based on the patients’ clinical course, TnT values may not have been representative, and ECGs were therefore reviewed for OMI classification (OMI cases N = 15).

### 2.4 Application of QoH and ECG Criteria

QoH was retrospectively applied to all cases after the adjudication process. Powerful Medical did not participate in the adjudication of OMI diagnoses. All ECGs were exported in xml format to Powerful Medical who were blinded to the classification results when applying the QoH software (aOMI v1) to the ECGs. The results of QoH were then transferred to Lund University for the final data analysis.

The ECG criteria described below were applied to all patients using the automated measurements, eg, ST-J, R-, and S-wave amplitudes provided by the Glasgow ECG analysis program, in which ST deviation is measured relative to the PQ junction. Manual measurements or physician over-reading were not performed. STEMI criteria were met if ST elevation ≥1 mm was present in at least 2 contiguous leads except V2-V3 where the following cutoffs are used instead: male ≥40 years old, 0.2 mV, <40 years old ≥0.25 mV, female ≥0.15 mV).^1^ For this, measurements from the automated analysis (Glasgow ECG analysis program (v.30.4.3)) were used, ie, no manual measurements or human interpretation were performed to determine whether STEMI criteria were met or not. Since STEMI criteria are not applicable to patients with left bundle branch block (LBBB), ventricular pacing and left ventricular hypertrophy (LVH),^1^ the analysis was also performed in a subset of the population without these ECG diagnoses.

To allow for a comparison between QoH and algorithmically measured ECG STEMI criteria, ie, even among cases for which conventional STEMI criteria are not applicable (LVH, ventricular pacing or LBBB), an extended algorithmic diagnostic approach was used. This approach included previously validated criteria for the detection of OMI in cases with LVH, LBBB and ventricular pacing.^24^^,^^25^^,^^30^, ^31^, ^32^ Cases with LBBB or ventricular pacing were identified based on the diagnostic statements provided by the Glasgow ECG analysis program (v.30.4.3). In cases with LBBB or ventricular pacing, ECG criteria for OMI were fulfilled if ST elevation occurred in leads with predominantly positive QRS complexes (concordant ST elevation), or if concordant ST depression (≥0.1 mV) was present in leads V1-V3 (LBBB) or V1-V6 (paced rhythm), or if ST elevation/S-wave amplitude was ≥0.25.^24^^,^^25^^,^^30^^,^^31^ LVH was considered present if the S-wave amplitude in V3 plus the R-wave amplitude in aVL exceeded >2.8 mV in males or >2.0 mV in females.^33^ In cases with LVH, the ECG criteria for OMI were fulfilled if ST elevation was equal or exceeded 25% of the summed R- and S-wave amplitudes in leads V1-V3, or in one of these leads if T-wave inversion was present.^32^

Additionally, to overcome the anatomical limitation of the 12-lead ECG regarding OMI detection, eg, in isolated posterior infarction,^34^^,^^35^ we also included an extension of STEMI criteria in which additional lead pairs are assessed for presence of ST elevation (-III/aVL, II/-aVR, -aVR/I, III/-aVL, -V1/-V2, and -V2/-V3).^14^^,^^34^ This means that the ECG was classified as positive for OMI in case of ST elevation in -V2 and -V3, or III and -aVL. Including these additional leads in OMI detection has been described to enhance sensitivity without any loss of specificity.^34^

The inclusion of specific criteria for LBBB, ventricular pacing and LVH, in combination with the additional leads will hereafter be referred to as “Extended STEMI criteria.”

In addition, the diagnostic accuracy of the diagnostic statements provided by the Glasgow ECG analysis program (v.30.4.3) regarding AMI was also evaluated, hereafter referred to as “Computer statement.” Statements were first classified as being either a) strongly indicative of AMI, b) suggestive of AMI, or c) related to myocardial ischemia, see definitions in Tables S1-S3. For the primary analysis, statements classified as either strongly indicative or suggestive of AMI were used to define a positive Computer statement regarding OMI. In the Receiver Operating Curve (ROC) analysis described below, all 3 levels of confidence of the computer statements were included.

### 2.5 Statistical Analysis

Continuous variables were described as mean with standard deviation, or as median with interquartile range. Proportional differences between groups were assessed using the χ^2^ test. Comparison of group means was performed using Student’s *t*-test or analysis of variance. Sensitivity, specificity, accuracy (true positives + true negatives)/(positives and negatives), positive/negative predictive values, positive/negative likelihood ratio (LR+, LR−) for the detection of OMI were calculated for QoH, STEMI criteria the extended STEMI criteria and the Computer statement. In addition, diagnostic odds ratio was calculated (true positives × true negatives)/(false positives × false negatives).^36^ The output of the QoH model is numerical on a scale from 0-1.0; the threshold of >0.5 has been optimized for sensitivity and specificity in the intended use population (those with suspicion of ACS). Since QoH results can either be presented as a binary output (positive/negative for OMI if > or <0.50) or as a continuous result ranging from 0 to 1, the diagnostic accuracy was also presented based on different cutoffs for the continuous output, both numerically and through ROC analysis with area under the curve (AUC). The association between the continuous QoH output was also described as an odds ratio (OR) for each 0.1 unit increase, based on logistic regression analysis with OMI as the outcome variable. Sensitivity analyses were performed to describe the diagnostic accuracy of the different methods with different subsets of patients and different definitions for OMI classifications, eg, considering only patients who underwent coronary angiography, using 100% occlusion for OMI only, including PCI but not CABG as a relevant intervention only, and are presented in the Supplements (Table S4). All statistical analyses were performed using R, version 4.3.1 (R Core Team, 2023. *R: A Language and Environment for Statistical Computing*. R Foundation for Statistical Computing, Vienna, Austria. URL: https://www.R-project.org/). A two-sided *P* value < .05 was considered statistically significant.

## 3 Results

In the 20-month study period, 26,545 unique patients presented at the 5 EDs with a primary complaint of nontraumatic chest pain. Of these, 2032 patients had either no ECG recorded or an ECG of unacceptable quality, and 2 were missing register data for reliable OMI adjudication (incorrect Social Security number recorded, missing angiographic data). After exclusions, 24,511 patients remained. Baseline characteristics are presented in Table 1. Among the 24,511 patients, 1589 AMI occurred, among which 467 (31%) were classified as OMI, yielding an OMI prevalence of 1.9% in the studied population. Patient outcomes stratified by QoH results are presented in Table 2. Based on International Classification of Diseases (ICD) codes, 199 (43%) of the patients with OMI received a STEMI diagnosis, 206 (44%) an NSTEMI diagnosis, and the remaining 62 patients an unspecified AMI. Among patients with a STEMI ICD diagnosis, 71% were classified as OMI in the adjudication process, and the corresponding proportion among NSTEMI patients was 20%. Patients with OMI were older, more often male, and more often had previous ischemic heart disease, including previous AMI.

**Table 1:** Baseline characteristics.

	All	QoH negative	QoH positive
n	24,511	24,031	480
Age, years	59 ± 19	59 ± 19	70 ± 14
Troponin T, ng/L	7 (4-17)	7 (4-16)	991 (151-2828)
Male sex, n (%)	12,708 (51.8)	12,386 (51.5)	324 (67.5)
*Medical history*			
IHD, n (%)	3624 (14.8)	3516 (14.6)	109 (22.7)
Prior PCI, n (%)	1417 (5.8)	1369 (5.7)	48 (10.0)
Prior CABG, n (%)	392 (1.6)	374 (1.6)	18 (3.8)
Previous AMI, n (%)	1491 (6.1)	1437 (6.0)	54 (11.2)
Angina pectoris, n (%)	1587 (6.5)	1545 (6.4)	42 (8.8)
Unstable angina, n (%)	546 (2.2)	531 (2.2)	15 (3.1)
Heart failure, n (%)	1456 (5.9)	1413 (5.9)	43 (9.0)
Diabetes, n (%)	2086 (8.5)	2032 (8.5)	54 (11.2)
Hypertension, n (%)	5015 (20.5)	4896 (20.4)	120 (25.0)
Pulmonary embolism, n (%)	302 (1.2)	294 (1.2)	8 (1.7)
Cerebrovascular diseases, n (%)	890 (3.6)	869 (3.6)	21 (4.4)
COPD, n (%)	925 (3.8)	905 (3.8)	20 (4.2)
Renal failure, n (%)	609 (2.5)	589 (2.5)	20 (4.2)
PAD, n (%)	376 (1.5)	364 (1.5)	12 (2.5)
*Medications*			
Antithrombotics, n (%)	5721 (23.3)	5567 (23.2)	154 (32.1)
Statin, n (%)	6685 (27.3)	6524 (27.2)	161 (33.5)
Antihypertensive, n (%)	8969 (36.6)	8733 (36.3)	235 (49.0)
Betablocker, n (%)	7374 (30.1)	7197 (30.0)	176 (36.7)
Anticoagulants, n (%)	1321 (5.4)	1300 (5.4)	21 (4.4)
Insulin, n (%)	1393 (5.7)	1346 (5.6)	47 (9.8)
Diuretics, n (%)	3621 (14.8)	3530 (14.7)	91 (19.0)
ACE/AII-antagonists, n (%)	7626 (31.1)	7426 (30.9)	199 (41.5)

ACE/AII, angiotensin converting enzyme/angiotensin II; AMI, acute myocardial infarction; CABG, coronary artery bypass grafting; COPD, chronic obstructive pulmonary disease; IHD, ischemic heart disease; OMI−, negative for OMI according to Queen of Hearts, ie, either nonocclusion myocardial infarction or no acute myocardial infarction; OMI+, positive for OMI according to Queen of Hearts; PAD, peripheral artery disease; PCI, percutaneous coronary intervention.

Data are presented as mean ± standard deviation for age, median (interquartile range) for troponin T, and n (%) for categorical variables.

**Table 2:** Outcomes described for the whole cohort (n = 24,513) and stratified based on the results of Queen of Hearts.

n	All	QoH−	QoH+	*P*
	24,513	24,033	480	
AMI	1591 (6.5)	1215 (5.1)	376 (78.3)	<.001
OMI, n (%)	467 (1.9)	224 (0.9)	243 (50.6)	<.001
NOMI, n (%)	1122 (4.6)	989 (4.1)	133 (27.7)	<0.001
Coronary angiography, n (%)	2475 (10.1)	2126 (8.8)	349 (72.7)	<.001
PCI	1490 (6.1)	1205 (5.0)	285 (59.4)	<.001
CABG	275 (1.1)	251 (1.0)	24 (5.0)	<.001

AMI, acute myocardial infarction; CABG, coronary artery bypass grafting; NOMI, nonocclusion myocardial infarction; OMI, occlusion myocardial infarction; PCI, percutaneous coronary intervention; QoH−, negative result of Queen of Hearts; QoH+, positive result of Queen of Hearts.

### 3.1 Diagnostic Accuracy

Measures of diagnostic accuracy of each ECG classifier are presented in Table 3. For OMI identification, sensitivity was highest for QoH (52% [47-57]), followed by the extended STEMI criteria (41% [36-45]). QoH provided the highest PPV (51% [47-54]), followed by the computer statement (26% [23-30]). QoH increased sensitivity by 29 percentage points (22-35) compared with STEMI criteria, 12 percentage points (5-19) compared with extended STEMI criteria, and 21 percentage points (14-27) compared with the Computer statement.

**Table 3:** Diagnostic accuracy for detection of occlusion myocardial infarction (OMI) for Queen of Hearts (QoH), STEMI criteria, the extended STEMI criteria and the computer statement.

Full data set, N = 24,511, 467 OMI cases							DOR
	Sensitivity, %	Specificity, %	PPV, %	NPV, %	LR+	LR−	
QoH	52 [47-57]	99 [99-99]	51 [47-54]	99 [99-99]	53 [45-62]	0.48 [0.44-0.53]	110
STEMI criteria	23 [1-27]	98 [98-98]	17 [15-20]	98 [98-99]	11 [9.0-13]	0.79 [0.75-0.83]	14
Extended STEMI criteria	41 [36-45]	95 [95-96]	14 [12-15]	99 [99-99]	8.3 [7.3-9.3]	0.62 [0.58-0.67]	14
Computer statement	32 [28-37]	98 [98-98]	26 [23-30]	99 [99-99]	19 [23-30]	0.69 [0.65-0.74]	28
*Subset of the data including only patients for which STEMI criteria are applicable, N = 21,972, 411 OMI cases*							
QoH	55 [58-59]	99 [99-99]	52 [48-56]	99 [99-99]	58 [49-68]	0.46 [0.41-0.51]	126
STEMI criteria	26 [22-31]	98 [97-98]	17 [15-20]	99 [98-99]	11 [9.2-13]	0.76 [0.72-0.80]	14
Extended STEMI criteria	42 [38-47]	95 [95-96]	15 [13-16]	99 [99-99]	8.9 [7.9-10]	0.61 [0.56-0.66]	15
Computer statement	33 [29-38]	98 [98-99]	28 [25-32]	99 [99-99]	21 [17-25]	0.68 [0.63-0.73]	31

DOR, diagnostic odds ratio; LR+, positive likelihood ratio; LR−, negative likelihood ratio; NPV, negative predictive value; PPV, positive predictive value; STEMI, ST-Elevation Myocardial Infarction.

Specificity and NPV were high for all methods (98% to 99%), but lowest for the extended STEMI criteria (95 [95 to 96]). LR+ was high for all methods, but highest for QoH (53 [45-62]), followed by the computer statement (19 [23-30]). The diagnostic OR was highest for QoH (109 [87-136]), followed by the Computer statement (27 [21-33]), STEMI criteria (14 [11-17]) and extended STEMI criteria (13 [11-16]).

**Figure 2:**
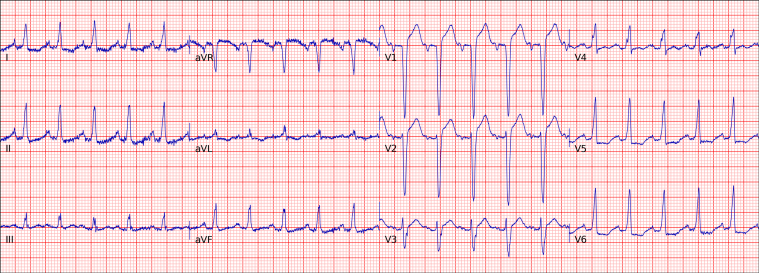
ECG from a 68-year-old female patient with OMI (troponin 4820 ng/L), which was negative by both STEMI criteria and extended STEMI criteria but positive according to QoH (0.93). The computer statement read “End QRS notching/slurring—early repolarization pattern.”

**Figure 3:**
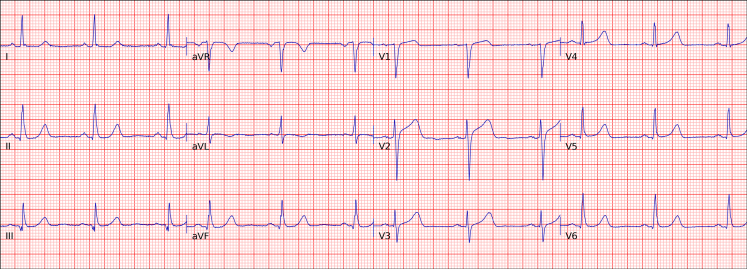
ECG from a 62-year-old male patient with OMI (troponin 1326 ng/L), which was positive by STEMI criteria and extended STEMI criteria but negative according to QoH (0.49). The computer statement read “Septal ST elevation, consider acute infarct.” Widespread ST-T abnormality may be due to myocardial ischemia.

Among the 360 patients with OMI who did not meet STEMI criteria, QoH correctly assigned 143 (40%) as OMI. Conversely, among the 23,540 patients without OMI, who were negative by STEMI criteria, QoH incorrectly classified 188 (0.8%) as OMI. Among patients classified as NOMI, QoH provided a false positive result, ie, indicating OMI, in 12% of cases, STEMI criteria in 0.1%, the extended STEMI criteria in 13%. and the computer statement in 8%. ECG examples are presented in Figs 2 and 3.

In the sensitivity analyses, QoH consistently showed higher sensitivity than STEMI criteria, extended STEMI criteria, and Computer statement.

**Figure 4:**
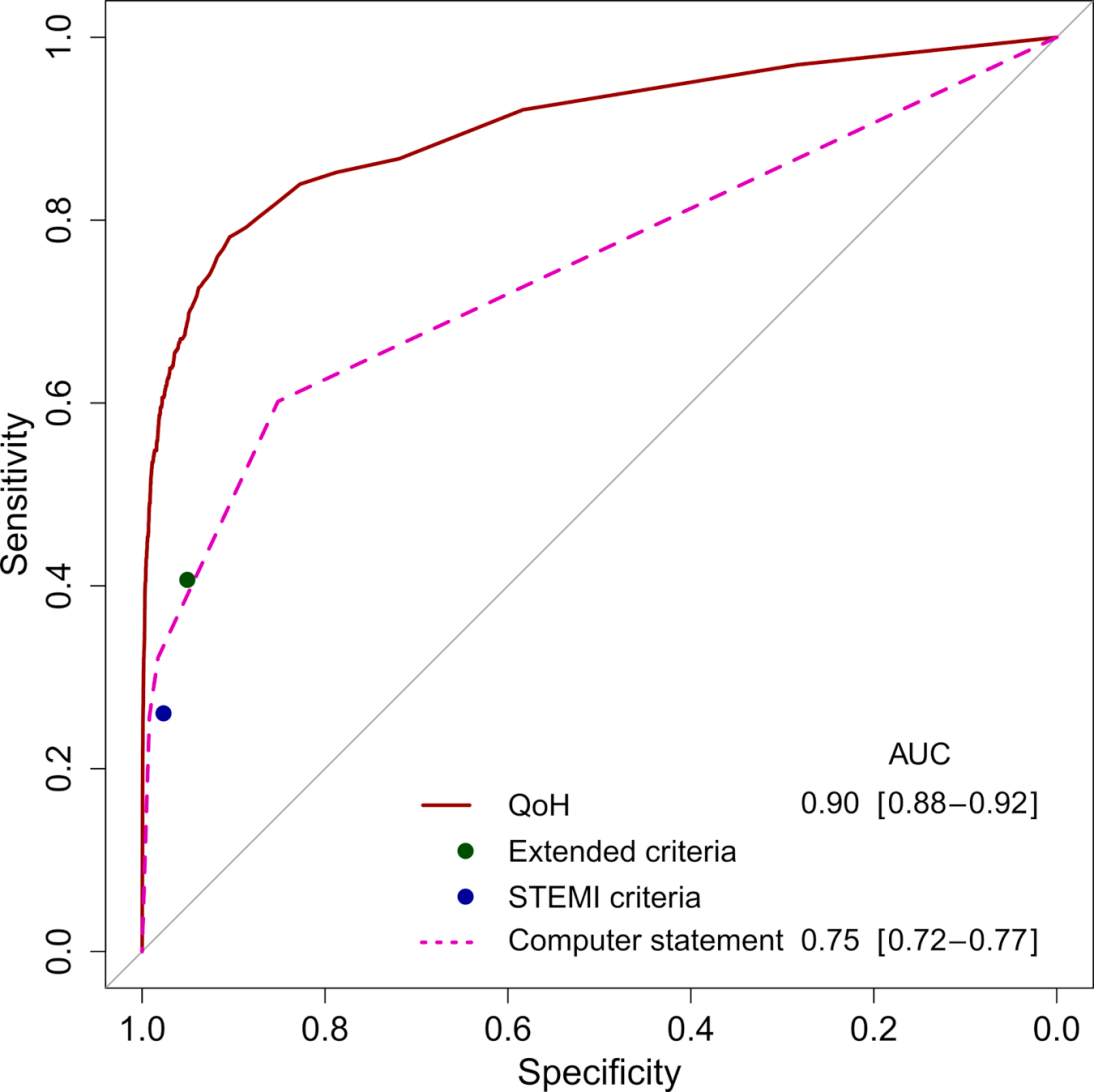
Diagnostic accuracy for Queen of Hearts (QoH), STEMI criteria, extended STEMI criteria and the computer statements described as area under the curve (AUC) with 95% confidence intervals. For the computer statements, 3 levels of certainty define the ROC curve (statements strongly indicative of acute myocardial infarction, statements suggestive of acute myocardial infarction, and statements related to myocardial ischemia).

Continuous QoH results, ranging from 0 to 1, were associated with increased odds of OMI, OR per each 0.1 increase: 1.32 (1.27-1.36). Higher QoH cutoffs increased PPV but slightly lowered NPV and vice versa for lower QoH cutoffs, Table 4. The AUC for the continuous QoH results was 0.90 (0.88-0.92), Fig 4. In the subset of patients with either LVH, LBBB or paced rhythm (n = 2502, 56 OMI cases), AUC was lower (0.82 [0.76-0.89]).

**Table 4:** Diagnostic accuracy of the Queen of Hearts presented for different cutoffs of the Queen of Hearts output (0 to 1).

Threshold	OMI+ (n = 467)		OMI− (n = 24,044)		Sens	Spec	Acc	PPV	NPV
	TP	FN	FP	TN					
>0.05	300	167	818	23,226	64.0	96,7	96,1	27,5	99,3
>0.10	284	183	585	23,459	60.8	97,6	96,9	33,0	99,2
>0.15	278	189	494	23,550	59.5	98,0	97,3	36,4	99,2
>0.20	268	199	428	23,616	57.4	98,2	97,5	38,8	99,2
>0.25	260	207	384	23,660	55.9	98,4	97,6	40,0	99,1
>0.30	256	211	352	23,692	54.8	98,6	97,8	43,8	99,1
>0.35	254	213	315	23,729	54.4	98,7	97,9	44,8	99,1
>0.40	251	216	29	23,754	53.7	98,8	97,9	46,4	99,1
>0.45	249	218	263	23,781	53.1	98,9	98,1	49,0	99,1
>0.50^a^	243	224	237	23,807	52.0	99,0	98,1	50,8	99,1
>0.55	235	232	217	23,827	50.3	99,1	98,2	52,1	99,0
>0.60	229	238	202	23,842	49.4	99,2	98,2	53,6	99,0
>0.65	220	247	178	23,866	47.7	99,2	98,3	56,1	99,0
>0.70	214	253	162	23,882	44.2	99,2	98,0	58,1	99,0
>0.75	210	257	141	23,903	41.5	99,2	98,0	58,7	99,0
>0.80	204	263	122	23,922	38.9	99,3	97,9	58,4	98,9
>0.85	199	268	106	23,938	36.3	99,3	97,8	58,2	98,8
>0.90	189	278	86	23,958	33.6	99,4	97,7	57,9	98,7
>0.95	158	309	60	23,984	31.1	99,4	97,5	57,5	98,6

Acc, accuracy; FN, false negatives; FP, false positives; NPV, negative predictive value; OMI, occlusion myocardial infarction; PPV, positive predictive value; sens, sensitivity, spec, specificity; TN, true negatives; TP, true positives.

a >0.5 is the default cutoff for Queen of Hearts and its results are highlighted in bold font.

### 3.2 Time to Coronary Angiography and Mortality

Among the 2475 patients who underwent coronary angiography, 426 (12.0%) had OMI. Among 243 patients with OMI who were positive according to QoH, only 21 (9.6%) had coronary angiography within 90 minutes, Table 5.

**Table 5:** Time delay between ECG and coronary angiography based on presence of occlusion or nonocclusion myocardial infarction stratified by results of the Queen of Hearts.

	Time (hours) from ECG to coronary angiography		Coronary angiography within 90 min from ECG, n (%)
	OMI−	OMI+	OMI+
QoH−	38 (22-69)	19 (7-33)	3 (1.6)
QoH+	20 (6-44)	2.8 (2.1-4.9)	21 (9.9)
Extended STEMI criteria−	38 (22-68)	14 (4.2-28)	8 (3.3)
Extended STEMI criteria+	27 (9.5-64)	2.8 (2.1-6.9)	17 (9.8)
STEMI criteria−	37 (22-68)	13 (3.9-28)	14 (4.2)
STEMI criteria+	13 (3.7-49)	2.3 (1.8-3.1)	11 (12)
Computer statement−	38 (22-69)	14 (4.2-30)	1 (0.9)
Computer statement+	24 (7.9-48)	2.7 (2.0-4.2)	15 (11)

OMI, occlusion myocardial infarction; QoH, Queen of Hearts.

Time to coronary angiography is described as median (interquartile range).

+/− refers to either a positive test result (QoH, extended STEMI criteria, STEMI criteria) or a positive/negative OMI diagnosis; STEMI, ST-Elevation Myocardial Infarction.

## 4 Limitations

In a retrospective design, correct classification of OMI cases is challenging, since coronary angiography is performed and reported based on clinical decision-making. Therefore, some OMI patients who did not undergo angiography may have been misclassified as NOMI. Also, the SCAAR register has no data on TIMI flow. The lack of TIMI flow data in the SCAAR registry is a limitation, and for pragmatic reasons, all 1323 angiographic films were not reviewed. Since many coronary arteries that are occluded at the time of the ECG are open by the time of angiography,^37^^,^^38^ restricting the definition of OMI to lower TIMI flow values will inflate sensitivity and deflate specificity, whereas the opposite will occur if higher TIMI flow values are included. Although this limits the numerical diagnostic accuracy reported in this study, it should have little effect on the comparison between methods, and the sensitivity analyses showed consistent superiority of QoH compared with other diagnostic methods. We presumed that acute coronary occlusion would be noted in SCAAR in cases with TIMI 0 flow, and possibly in some cases with TIMI 1. To minimize the number of missed OMI cases with TIMI 1, we used TnT information and reviewed angiography reports and films in specific patient cases. However, peak TnT are not registered in clinical routine, and the recorded levels, in many instances, may not reflect the peak levels. Also, in the registry data, chronic occlusions cannot be readily differentiated from acute occlusions, and chronic occlusions have been described to occur in 6% of patients with obstructive coronary artery disease.^39^ Presence of chronic occlusions can lead to misclassification of OMI but all patients with AMI and complete occlusions underwent PCI, suggesting a low risk of misclassification.

Both the prevalence of OMI cases (1.9%) and the proportion of all AMI cases which were OMI (29%) were low. The low prevalence is comparable to previous studies among Swedish EDs.^14^ In registry data, STEMI accounts for 27% of AMI diagnoses, and more than one third of NSTEMI can be classified as OMI.^4^^,^^40^ Therefore, OMI can be expected to account for about half of all AMIs. The lower OMI prevalence can, in part, be explained by the large proportion of STEMI patients being directed to the cath lab before ED arrival, leaving a lower number of STEMI cases at the ED. Since this is often the case,^14^ the ED chest pain population may represent a diagnostically challenging population for clinicians and AI-based ECG interpretation models.

The retrospective design makes the true clinical impact of the diagnostic accuracy reported in this study challenging to decipher. For example, most patients with false positive STEMI criteria were not—and in most cases correctly so—managed as STEMI, as indicated by the low proportion of patients with AMI and the low proportion undergoing coronary angiography. This suggests a need for prospective studies determining the impact of AI models on patients outcomes. Such data have the potential to increase acceptance of the use of AI technologies among clinical practitioners.

The ECGs analyzed in this study were obtained as part of routine care for patients presenting with chest pain at the ED. Consequently, the cohort likely included some individuals for whom OMI was an unlikely diagnosis. Also, fulfillment of STEMI criteria was based on the measurements obtained by the ECG software, ie, manual expert reading was not performed. Automated measurements of ST deviation have been shown to be smaller than those manually measured.^41^ Thus, human measurements would likely have had an effect of higher sensitivity and lower specificity, but human interpretation may also have had the possibility to include other ECG findings to either suspect or dismiss OMI. Additionally, the study lacks information about the quality and duration of the chest pain, and there was no differentiation between typical, atypical, or non-anginal pain, which is a limitation. This likely resulted in a lower pre-test probability than when a clinician suspects OMI, and given the high LR+, a higher PPV for QoH would be expected in settings with higher prevalence of OMI.

## 5 Discussion

The main finding of this study was that, among ED chest pain patients, an AI ECG-model, QoH, increased the number of detected OMI compared to the established STEMI criteria, our extended STEMI criteria approach, and Computer statements without any loss in specificity or NPV. Our findings thus support previous reports of improved diagnostic accuracy of QoH compared with conventional ECG criteria.^9^^,^^16^ The findings also indicate that current STEMI criteria are insufficient for OMI detection in any setting, but particularly in settings with low prevalence of OMI, which has been shown previously.^3^^,^^13^^,^^14^ Compared to the previous external validation in 633 patients,^16^ sensitivity was lower for both QoH and STEMI criteria in the present study. This may be explained by the exclusion of more typical STEMI cases in the present study, since these were identified in the ambulance and bypassed the ED. Our OMI patients might therefore have had less typical ECG changes. The results of this study thus provide further strength to previous external validation of QoH and indicate a potential improvement over currently recommended ECG criteria.

Patients with OMI and a positive QoH had shorter time to coronary angiography compared with patients with either negative QoH or those without OMI. This suggests that physicians likely interpreted these ECGs as indicative of ischemia or that the clinical condition of these patients warranted early coronary angiography. However, only a minority of these patients underwent coronary angiography within 90 minutes, highlighting the potential to reduce the time from diagnosis to PCI in patients with OMI if QoH were utilized as a diagnostic tool.

Several AI models have been created for patients with a suspicion of ACS, but only a few models were explicitly trained to detect OMI.^15^^,^^16^^,^^42^, ^43^, ^44^, ^45^, ^46^ Goto et al^45^ derived an AI model to identify ED patients with chest pain in need of urgent catheterization from ∼40,000 cases. Based on ECG alone, the model achieved an AUC of 0.89 (0.84-0.92). However, the model does not provide information on the occlusion status of the coronary vessels, and has not been externally validated. Based on the prehospital ECG and information on age and sex, Al-Zaiti et al^44^ developed a machine learning-based prediction model which increased sensitivity for detecting ACS compared with expert ECG readers, despite including ECG patterns (bundle branch block, ventricular pacing) known to complicate the identification of ACS. However, specificity of the model /76%) was lower than for expert readers (94%). Al-Zaiti et al^15^ also recently described an AI model trained on 4026 patients’ prehospital ECGs, for the detection of OMI, excluding prehospital STEMI. During external validation, this model achieved high diagnostic accuracy (AUC: 0.87 [0.85-0.90]), and the model outperformed practicing clinicians as well as computer-based interpretations. In future studies, a head-to-head comparison of QoH and the model described by Al-Zaiti et al seems warranted.

Using the same database as in the present study, Nyström et al,^47^ developed a model based on ECG features, patient history, point-of-care laboratory values (hemoglobin, glucose, creatinine) and hs-TnT values. This model achieved higher diagnostic accuracy (0.95 [0.94 to 0.97]) than QoH, but the latter is based on ECG information only.

In our study, the diagnostic accuracy of the computer statement was lower than previously reported. Bosson et al^48^ reported that the Glasgow program had higher sensitivity and PPV for cases needing emergent coronary angiography when the software reported that the ECG met STEMI criteria. Bosson et al’s study thereby used a different endpoint (not OMI) than ours, and included patients with a prehospital triage directly to the cath lab. Importantly, in contrast to QoH, the Glasgow program, is not designed specifically to identify patients with OMI.

In this retrospective diagnostic accuracy study of ED patients with chest pain, QoH showed improved sensitivity in OMI detection compared with currently available ECG criteria, with similar specificity.

## Author Contributions

Thomas Lindow: Conceptualization, Methodology, Formal analysis, Visualization, Writing - Original Draft; Axel Nyström: Methodology, Data curation, Software, Writing - Review & Editing; Jakob Lundager Forberg: Conceptualization, Methodology, Writing - Review & Editing; Arash Mokhtari: Conceptualization, Methodology, Investigation, Writing - Review & Editing; Anders Björkelund: Data curation, Software, Writing - Review & Editing; Robert Herman: Software, Writing - Review & Editing; H Pendell Meyers: Methodology, Writing - Review & Editing; Stephen W. Smith: Methodology, Writing - Review & Editing; Ulf Ekelund: Conceptualization, Methodology, Funding Acquisition, Project administration, Writing - Review & Editing, Supervision.

## Funding and Support

This study was supported by an ALF research grant at Skåne University Hospital, by a grant from Region Skåne and Region Kronoberg, and by a grant from the Swedish Heart-Lung Foundation. This study was part of the AIR Lund (Artificially Intelligent use of Registers at Lund University) research environment and received funding from the Swedish Research Council (VR; grant no. 2019-00198). Funding organizations had no role in the planning, design, or conduct of the study, collection, analysis or interpretation of data, or preparation, review, or approval of the manuscript. Powerful Medical did not provide funding for this study.

## Conflict of Interest

SWS: Shareholder of Powerful Medical; RH: Shareholder and Chief Medical Officer of Powerful Medical; HPM: Shareholder and consultant of Powerful Medical. SWS, HPM, and RH took part in the study design and interpretation of the data and critically revised the manuscript. Powerful Medical assisted with the application of QoH on all subjects. This was an academically initiated study, and Powerful Medical did not participate in the adjudication of OMI diagnoses and they were blinded to the classification results when applying the QoH software (aOMI v1) to the ECGs.
