# Supplementary material for: Improved Detection of Acute Coronary Occlusion Myocardial Infarction by an Artificial Intelligence Electrocardiogram Model in Swedish Emergency Departments
Source: J Am Coll Emerg Physicians Open. 2026 Aug 14;7(5):100473. doi: 10.1016/j.acepjo.2026.100473 (PMC13482599; doi:10.1016/j.acepjo.2026.100473)
Supplement: Supplementary file 1 [file mmc1.docx]

**APPENDIX**

**Table S1.** Computer statements strongly indicative of acute myocardial infarction

*** ANTERIOR INFARCT - POSSIBLY ACUTE ***

*** ANTEROLATERAL INFARCT - POSSIBLY ACUTE ***

*** ANTEROSEPTAL INFARCT - POSSIBLY ACUTE ***

*** EXTENSIVE INFARCT - POSSIBLY ACUTE ***

*** INFERIOR INFARCT - POSSIBLY ACUTE ***

*** LATERAL INFARCT - POSSIBLY ACUTE ***

*** SEPTAL INFARCT - POSSIBLY ACUTE ***

Anterior ST elevation, CONSIDER ACUTE INFARCT

Anterolateral ST elevation, CONSIDER ACUTE INFARCT

Anteroseptal ST elevation, CONSIDER ACUTE INFARCT

Inferior ST elevation, CONSIDER ACUTE INFARCT

Inferior and ant/septal ST elevation, CONSIDER ACUTE INFARCT

Inferior and anterior ST elevation, CONSIDER ACUTE INFARCT

Inferior and lateral ST elevation, CONSIDER ACUTE INFARCT

Lateral ST elevation, CONSIDER ACUTE INFARCT

Marked anteroseptal ST depression, CONSIDER ACUTE INFARCT

Marked inferior ST depression, CONSIDER ACUTE INFARCT

Marked lateral ST depression, CONSIDER ACUTE INFARCT

Septal ST elevation, CONSIDER ACUTE INFARCT

Widespread ST depression, CONSIDER ACUTE INFARCT (left main occlusion / multivessel disease)

Widespread ST elevation, CONSIDER ACUTE INFARCT

**Table S2.** Computer statements suggestive of acute myocardial infarction

Ant/septal and lateral ST-T abnormality suggests myocardial infarct

Ant/septal and lateral ST-T abnormality suggests myocardial injury/ischemia

Ant/septal and lateral T wave abnormality suggests myocardial infarct

Anterior ST abnormality suggests myocardial injury/ischemia

Anterior ST-T abnormality suggests myocardial injury/ischemia

Anterior T wave abnormality suggests myocardial injury/ischemia

Anterolateral ST-T abnormality suggests myocardial injury/ischemia

Anterolateral T wave abnormality suggests myocardial infarct

Anteroseptal ST abnormality suggests myocardial injury/ischemia

Anteroseptal ST depression is probably reciprocal to inferior infarct

Anteroseptal ST-T abnormality suggests myocardial infarct

Anteroseptal ST-T abnormality suggests myocardial injury/ischemia

Anteroseptal T wave abnormality suggests myocardial injury/ischemia

Inferior ST abnormality suggests myocardial injury/ischemia

Inferior T wave abnormality is age and gender related

Inferior T wave abnormality suggests myocardial infarct

Inferior and ant/septal ST abnormality suggests myocardial injury/ischemia

Inferior and ant/septal ST-T abnormality suggests myocardial infarct

Inferior and ant/septal ST-T abnormality suggests myocardial injury/ischemia

Inferior and anterior ST-T abnormality suggests myocardial injury/ischemia

Inferior/lateral ST-T abnormality suggests myocardial infarct

Inferior/lateral ST-T abnormality suggests myocardial injury/ischemia

Lateral ST abnormality suggests myocardial injury/ischemia

Lateral ST-T abnormality suggests myocardial injury/ischemia

Marked anteroseptal ST depression accompanies the infarct

Marked inferior ST depression accompanies the infarct

Marked lateral ST depression accompanies the infarct

Septal ST abnormality suggests myocardial injury/ischemia

Septal ST-T abnormality suggests myocardial injury/ischemia

Septal and lateral ST-T abnormality suggests myocardial injury/ischemia

Tall T waves - consider acute ischemia or hyperkalemia

Widespread ST abnormality suggests myocardial injury/ischemia

Widespread ST-T abnormality suggests myocardial infarct

Widespread ST-T abnormality suggests myocardial injury/ischemia

Widespread T wave abnormality suggests myocardial infarct

**Table S3.** Computer statements related to myocardial ischemia

Ant/septal and lateral ST abnormality may be due to myocardial ischemia

Ant/septal and lateral ST abnormality may be due to the hypertrophy and/or ischemia

Ant/septal and lateral ST-T abnormality may be due to myocardial ischemia

Ant/septal and lateral ST-T abnormality may be due to the hypertrophy and/or ischemia

Ant/septal and lateral T wave abnormality may be due to myocardial ischemia

Ant/septal and lateral T wave abnormality may be due to the hypertrophy and/or ischemia

Anterior ST abnormality may be due to myocardial ischemia

Anterior T wave abnormality may be due to myocardial ischemia

Anterior T wave abnormality may be due to the hypertrophy and/or ischemia

Anterolateral ST abnormality may be due to myocardial ischemia

Anterolateral ST abnormality may be due to the hypertrophy and/or ischemia

Anterolateral ST elevation - cannot rule out myocardial injury

Anterolateral ST-T abnormality may be due to myocardial ischemia

Anterolateral ST-T abnormality may be due to the hypertrophy and/or ischemia

Anterolateral T wave abnormality may be due to myocardial ischemia

Anterolateral T wave abnormality may be due to the hypertrophy and/or ischemia

Anteroseptal ST abnormality may be due to myocardial ischemia

Anteroseptal ST abnormality may be due to the hypertrophy and/or ischemia

Anteroseptal ST-T abnormality may be due to myocardial ischemia

Anteroseptal ST-T abnormality may be due to the hypertrophy and/or ischemia

Anteroseptal T wave abnormality may be due to myocardial ischemia

Anteroseptal T wave abnormality may be due to the hypertrophy and/or ischemia

Cannot rule out anterior infarct - age undetermined

Cannot rule out anteroseptal infarct - age undetermined

Cannot rule out septal infarct - age undetermined

Extensive infarct - age undetermined

Inferior ST abnormality may be due to myocardial ischemia

Inferior ST abnormality may be due to the hypertrophy and/or ischemia

Inferior ST elevation - cannot rule out myocardial injury

Inferior ST-T abnormality may be due to myocardial ischemia

Inferior ST-T abnormality may be due to the hypertrophy and/or ischemia

Inferior T wave abnormality may be due to myocardial ischemia

Inferior T wave abnormality may be due to the hypertrophy and/or ischemia

Inferior and ant/septal ST abnormality may be due to myocardial ischemia

Inferior and ant/septal ST abnormality may be due to the hypertrophy and/or ischemia

Inferior and ant/septal ST-T abnormality may be due to myocardial ischemia

Inferior and ant/septal ST-T abnormality may be due to the hypertrophy and/or ischemia

Inferior and ant/septal T wave abnormality may be due to myocardial ischemia

Inferior and anterior ST-T abnormality may be due to myocardial ischemia

Inferior and anterior ST-T abnormality may be due to the hypertrophy and/or ischemia

Inferior and anterior T wave abnormality may be due to myocardial ischemia

Inferior and anterior T wave abnormality may be due to the hypertrophy and/or ischemia

Inferior and septal ST-T abnormality may be due to myocardial ischemia

Inferior and septal ST-T abnormality may be due to the hypertrophy and/or ischemia

Inferior and septal T wave abnormality may be due to the hypertrophy and/or ischemia

Inferior/lateral ST abnormality may be due to myocardial ischemia

Inferior/lateral ST abnormality may be due to the hypertrophy and/or ischemia

Inferior/lateral ST-T abnormality may be due to myocardial ischemia

Inferior/lateral ST-T abnormality may be due to the hypertrophy and/or ischemia

Inferior/lateral T wave abnormality may be due to myocardial ischemia

Inferior/lateral T wave abnormality may be due to the hypertrophy and/or ischemia

Lateral ST abnormality may be due to myocardial ischemia

Lateral ST abnormality may be due to the hypertrophy and/or ischemia

Lateral ST elevation - cannot rule out myocardial injury

Lateral ST-T abnormality may be due to myocardial ischemia

Lateral ST-T abnormality may be due to the hypertrophy and/or ischemia

Lateral T wave abnormality may be due to myocardial ischemia

Lateral T wave abnormality may be due to the hypertrophy and/or ischemia

Septal ST abnormality may be due to myocardial ischemia

Septal ST abnormality may be due to the hypertrophy and/or ischemia

Septal ST-T abnormality may be due to myocardial ischemia

Septal ST-T abnormality may be due to the hypertrophy and/or ischemia

Septal T wave abnormality may be due to the hypertrophy and/or ischemia

Septal and lateral ST abnormality may be due to myocardial ischemia

Septal and lateral ST-T abnormality may be due to myocardial ischemia

Septal and lateral ST-T abnormality may be due to the hypertrophy and/or ischemia

Septal and lateral T wave abnormality may be due to myocardial ischemia

Septal and lateral T wave abnormality may be due to the hypertrophy and/or ischemia

Widespread ST abnormality may be due to the hypertrophy and/or ischemia

Widespread ST-T abnormality may be due to myocardial infarct or CVA

Widespread ST-T abnormality may be due to myocardial ischemia

Widespread ST-T abnormality may be due to the hypertrophy and/or ischemia

Widespread T wave abnormality may be due to myocardial ischemia

Widespread T wave abnormality may be due to the hypertrophy and/or ischemia

| **Table S4. Sensitivity analyses describing the diagnostic accuracy for the Queen of Hearts, STEMI criteria, Extended STEMI criteria and computer statements** | | | | | | |
| --- | --- | --- | --- | --- | --- | --- |
|  | Considering 100% occlusion only as definitive OMI  (OMI cases: N=252) | | Considering only patients who underwent angiography (N=2474, OMI cases: N=427) | | Excluding patients with CABG (N=24,341, OMI cases: N=446) | |
|  | Sensitivity, % | Specificity, % | Sensitivity, % | Specificity, % | Sensitivity, % | Specificity, % |
| QoH | 38 | 99 | 51 | 94 | 54 | 99 |
| STEMI criteria | 17 | 98 | 23 | 97 | 24 | 98 |
| Extended STEMI criteria | 32 | 94 | 41 | 91 | 41 | 95 |
| Computer statement | 32 | 98 | 33 | 95 | 33 | 98 |

The STARD reporting checklist

For checking that diagnostic accuracy study articles can be understood and used by everyone

|  | Item Description | Location (or reason for not reporting) |
| --- | --- | --- |
| **Title or abstract** |  |  |
| [1. Identification as a study of diagnostic accuracy](https:/resources.equator-network.org/reporting-guidelines/stard/items/identification.html?utm_source=stard&utm_medium=checklist&utm_campaign=STARD_2015_v1_1) | Identification as a study of diagnostic accuracy using at least one measure of accuracy (such as sensitivity, specificity, predictive values, or AUC). | Abstract background and methods |
| **Abstract** |  |  |
| [2. Abstract](https:/resources.equator-network.org/reporting-guidelines/stard/items/abstract.html?utm_source=stard&utm_medium=checklist&utm_campaign=STARD_2015_v1_1) | Structured summary of study design, methods, results and conclusions (for specific guidance, see STARD for Abstracts). | Abstract |
| **Introduction** |  |  |
| [3. Background](https:/resources.equator-network.org/reporting-guidelines/stard/items/background.html?utm_source=stard&utm_medium=checklist&utm_campaign=STARD_2015_v1_1) | Scientific and clinical background, including the intended use and clinical role of the index test. | Page 2-3 |
| [4. Objectives](https:/resources.equator-network.org/reporting-guidelines/stard/items/objectives.html?utm_source=stard&utm_medium=checklist&utm_campaign=STARD_2015_v1_1) | Study objectives and hypotheses. | Page 5 (line 112) and p 6 (line 124) |
| **Methods** |  |  |
| [5. Study design](https:/resources.equator-network.org/reporting-guidelines/stard/items/study-design.html?utm_source=stard&utm_medium=checklist&utm_campaign=STARD_2015_v1_1) | Whether data collection was planned before the index test and reference standard were performed (prospective study) or after (retrospective study). | Page 3 |
| Participants |  |  |
| [6. Eligibility criteria](https:/resources.equator-network.org/reporting-guidelines/stard/items/eligibility-criteria.html?utm_source=stard&utm_medium=checklist&utm_campaign=STARD_2015_v1_1) | Eligibility criteria. | Page 3 |
| [7. Identifying eligible participants](https:/resources.equator-network.org/reporting-guidelines/stard/items/identifying-eligible-participants.html?utm_source=stard&utm_medium=checklist&utm_campaign=STARD_2015_v1_1) | On what basis potentially eligible participants were identified (such as symptoms, results from previous tests, inclusion in registry). | Page 3 |
| [8. Setting, location, and dates](https:/resources.equator-network.org/reporting-guidelines/stard/items/setting-location-dates.html?utm_source=stard&utm_medium=checklist&utm_campaign=STARD_2015_v1_1) | Where and when potentially eligible participants were identified (setting, location and dates). | Page 3 |
| [9. Consecutive, random or convenience series](https:/resources.equator-network.org/reporting-guidelines/stard/items/consecutive-random-convenience-series.html?utm_source=stard&utm_medium=checklist&utm_campaign=STARD_2015_v1_1) | Whether participants formed a consecutive, random or convenience series. | Page 3 |
| Test Methods |  |  |
| [10. Index test & Reference standard](https:/resources.equator-network.org/reporting-guidelines/stard/items/index-test.html?utm_source=stard&utm_medium=checklist&utm_campaign=STARD_2015_v1_1) | 10a. Index test  10b. Reference standard | Page 4 and 5 |
| [11. Reference standard rationale](https:/resources.equator-network.org/reporting-guidelines/stard/items/reference-standard-rationale.html?utm_source=stard&utm_medium=checklist&utm_campaign=STARD_2015_v1_1) | Rationale for choosing the reference standard (if alternatives exist). | Page 4 and 5 |
| [12. Index test and reference standard cut-offs or categories](https:/resources.equator-network.org/reporting-guidelines/stard/items/index-test-cut-offs-categories.html?utm_source=stard&utm_medium=checklist&utm_campaign=STARD_2015_v1_1) | 12a. Definition of and rationale for test positivity cut-offs or result categories of the index test, distinguishing prespecified from exploratory.  12b. Definition of and rationale for test positivity cut-offs or result categories of the reference standard, distinguishing prespecified from exploratory. | N/A |
| [13. Information available to performers or readers of the index test and reference standard assessors](https:/resources.equator-network.org/reporting-guidelines/stard/items/information-available-index.html?utm_source=stard&utm_medium=checklist&utm_campaign=STARD_2015_v1_1) | 13a. Whether clinical information and reference standard results were available to the performers or readers of the index test.  13b. Whether clinical information and index test results were available to the assessors of the reference standard. | Pages 5-6, lines 121 - 124 |
| Analysis |  |  |
| [14. Analysis methods](https:/resources.equator-network.org/reporting-guidelines/stard/items/analysis-methods.html?utm_source=stard&utm_medium=checklist&utm_campaign=STARD_2015_v1_1) | Methods for estimating or comparing measures of diagnostic accuracy. | Page 8, lines 178 - 190 |
| [15. Indeterminate results](https:/resources.equator-network.org/reporting-guidelines/stard/items/indeterminate-results.html?utm_source=stard&utm_medium=checklist&utm_campaign=STARD_2015_v1_1) | How indeterminate index test or reference standard results were handled. | Page 4 – OMI adjudication |
| [16. Missing data](https:/resources.equator-network.org/reporting-guidelines/stard/items/missing-data.html?utm_source=stard&utm_medium=checklist&utm_campaign=STARD_2015_v1_1) | How missing data on the index test and reference standard were handled. | N/A |
| [17. Variability](https:/resources.equator-network.org/reporting-guidelines/stard/items/variability.html?utm_source=stard&utm_medium=checklist&utm_campaign=STARD_2015_v1_1) | Any analyses of variability in diagnostic accuracy, distinguishing prespecified from exploratory. | N/A |
| [18. Intended sample size](https:/resources.equator-network.org/reporting-guidelines/stard/items/intended-sample-size.html?utm_source=stard&utm_medium=checklist&utm_campaign=STARD_2015_v1_1) | Intended sample size and how it was determined. | N/A |
| **Results** |  |  |
| Participants |  |  |
| [19. Participant flow diagram](https:/resources.equator-network.org/reporting-guidelines/stard/items/flow-diagram.html?utm_source=stard&utm_medium=checklist&utm_campaign=STARD_2015_v1_1) | Flow of participants, using a diagram. | Figure 1 |
| [20. Baseline characteristics](https:/resources.equator-network.org/reporting-guidelines/stard/items/baseline-characteristics.html?utm_source=stard&utm_medium=checklist&utm_campaign=STARD_2015_v1_1) | Baseline demographic and clinical characteristics of participants. | Table 1 |
| [21a. Participants with and without the target condition](https:/resources.equator-network.org/reporting-guidelines/stard/items/participants-with-target-condition.html?utm_source=stard&utm_medium=checklist&utm_campaign=STARD_2015_v1_1) | 21a. Distribution of severity of disease in those with the target condition.  21b. Distribution of alternative diagnoses in those without the target condition | Table 1 |
| [22. Time interval](https:/resources.equator-network.org/reporting-guidelines/stard/items/time-interval.html?utm_source=stard&utm_medium=checklist&utm_campaign=STARD_2015_v1_1) | Time interval and any clinical interventions between index test and reference standard. | N/A |
| Test Results |  |  |
| [23. Index test and reference standard results](https:/resources.equator-network.org/reporting-guidelines/stard/items/index-reference-results.html?utm_source=stard&utm_medium=checklist&utm_campaign=STARD_2015_v1_1) | Cross tabulation of the index test results (or their distribution) by the results of the reference standard. | Table 3 |
| [24. Estimates of accuracy](https:/resources.equator-network.org/reporting-guidelines/stard/items/estimates-of-accuracy.html?utm_source=stard&utm_medium=checklist&utm_campaign=STARD_2015_v1_1) | Estimates of diagnostic accuracy and their precision (such as 95% CIs). | Table 3 |
| [25. Adverse events](https:/resources.equator-network.org/reporting-guidelines/stard/items/adverse-events.html?utm_source=stard&utm_medium=checklist&utm_campaign=STARD_2015_v1_1) | Any adverse events from performing the index test or the reference standard. | N/A |
| **Discussion** |  |  |
| [26. Limitations](https:/resources.equator-network.org/reporting-guidelines/stard/items/limitations.html?utm_source=stard&utm_medium=checklist&utm_campaign=STARD_2015_v1_1) | Study limitations, including sources of potential bias, statistical uncertainty and generalisability. | p. 14 - 16, lines 321 - 369 |
| [27. Implications for Practice](https:/resources.equator-network.org/reporting-guidelines/stard/items/implications-for-practice.html?utm_source=stard&utm_medium=checklist&utm_campaign=STARD_2015_v1_1) | Implications for practice, including the intended use and clinical role of the index test. | p. 11 , lines 260 - 264 |
| **Other information** |  |  |
| [28. Registration](https:/resources.equator-network.org/reporting-guidelines/stard/items/registration.html?utm_source=stard&utm_medium=checklist&utm_campaign=STARD_2015_v1_1) | Registration number and name of registry. | Re-use of data from previous trial |
| [29. Protocol](https:/resources.equator-network.org/reporting-guidelines/stard/items/protocol.html?utm_source=stard&utm_medium=checklist&utm_campaign=STARD_2015_v1_1) | Where the full study protocol can be accessed. | No study protocol |
| [30. Funding](https:/resources.equator-network.org/reporting-guidelines/stard/items/funding.html?utm_source=stard&utm_medium=checklist&utm_campaign=STARD_2015_v1_1) | Sources of funding and other support; role of funders. | Title page |
